# Salivary NETosis-Related and Oxidative Stress Biomarkers Define a Conventional Cigarette Smoking-Associated Inflammatory Phenotype in Periodontitis: A Cross-Sectional Observational Study

**DOI:** 10.3390/biomedicines14061272

**Published:** 2026-06-02

**Authors:** Irina-Georgeta Sufaru, Luminita Lazar, Alexandra Cornelia Teodorescu, Norina Consuela Forna, Doriana Agop-Forna, Ana Petra Lazar, Maria Iacob, Teofana Amarie, Sorina Mihaela Solomon

**Affiliations:** 1 Grigore T. Popa University of Medicine and Pharmacy Iasi, 700115 Iasi, Romania; ursarescu.irina@umfiasi.ro (I.-G.S.); norina.forna@umfiasi.ro (N.C.F.); doriana.agop-forna@umfiasi.ro (D.A.-F.); iacob_maria@email.umfiasi.ro (M.I.); amarie.teofana@email.umfiasi.ro (T.A.); sorina.solomon@umfiasi.ro (S.M.S.); 2 George Emil Palade University of Medicine, Pharmacy, Science, and Technology of Targu Mures, Department of Periodontology, 540139 Targu Mures, Romania; 3 George Emil Palade University of Medicine, Pharmacy, Science, and Technology of Targu Mures, Department of Oral Rehabilitation and Occlusology, 540139 Targu Mures, Romania; ana.lazar@umfst.ro

**Keywords:** periodontitis, cigarette smoking, NETosis, salivary biomarkers, oxidative stress, MPO-DNA complexes

## Abstract

**Background/Objectives**: Cigarette smoking is a major risk factor for periodontitis, but the salivary host-response profile associated with smoking-related periodontal inflammation remains incompletely characterized. This study compared salivary NETosis-related and oxidative-inflammatory biomarkers among current smokers, former smokers, and never-smokers with periodontitis. **Methods:** This cross-sectional study included 159 systemically healthy adults with periodontitis (53 per group: current smokers, former smokers, never-smokers). Individuals with systemic diseases or concomitant medications that could interfere were excluded. Unstimulated whole saliva was analyzed for NETosis-related biomarkers (MPO-DNA complexes, citrullinated histone H3, neutrophil elastase, cell-free DNA) and oxidative-inflammatory markers (MMP-8, IL-1β, IL-6, TNF-α, 8-OHdG, total antioxidant capacity). **Results**: Salivary MPO-DNA complexes differed significantly among groups (current smokers 33.52 ± 9.96, former smokers 26.90 ± 8.38, never-smokers 19.20 ± 7.50 ng/mL; *p* < 0.001, *η*^2^ = 0.317). The composite NETosis score (*η*^2^ = 0.702) and oxidative-inflammatory score (*η*^2^ = 0.718) showed the same graded pattern. Biochemical verification confirmed clear group separation (salivary cotinine: current smokers 312.3 ± 77.0, former smokers 9.7 ± 5.1, never-smokers 3.2 ± 1.4 ng/mL). Smoking exposure was positively correlated with biomarker levels and the severity of periodontal disease. Smoking status remained independently associated with MPO-DNA complexes and the NETosis score after covariate adjustment. **Conclusions**: Current smoking was associated with an enhanced salivary NETosis-related and oxidative-inflammatory phenotype. Former smokers displayed an intermediate profile. Salivary MPO-DNA complexes and composite biomarker scores warrant further investigation as candidate non-invasive indicators of smoking-associated periodontal inflammatory burden, pending diagnostic performance analyses and prospective validation.

## 1. Introduction

Periodontitis is a chronic, biofilm-related, immune-inflammatory disease marked by the progressive destruction of tissues supporting the teeth, such as the periodontal ligament and alveolar bone [1]. While microbial dysbiosis is essential for initiating the disease, the extent and pattern of tissue loss are mainly influenced by the host’s inflammatory response [2]. Recent periodontal research has evolved from a solely microbial perspective to a more comprehensive approach that considers host susceptibility, systemic factors, immune cell activity, oxidative stress, and behavioral risks as collective contributors to disease development and progression [3,4].

Cigarette smoking is consistently recognized as a key modifiable risk factor for periodontitis. Epidemiological and clinical studies show that smokers tend to have more periodontal tissue destruction, greater clinical attachment loss, deeper pockets, compromised vascular and immune responses, and less favorable responses to periodontal therapy than non-smokers [5,6,7,8,9]. A recent systematic review confirms that smoking significantly increases the risk of developing periodontitis, underscoring its role as a major preventable factor in the burden of periodontal disease [10]. More recent biomarker-based meta-analytic evidence highlights the complex biological relationship between smoking, periodontal inflammation, and alveolar bone loss. This relationship is likely influenced by variables such as age, sex, systemic health, oral hygiene, and local inflammatory mediators, rather than tobacco exposure alone [11].

Cigarette smoking contributes to periodontal breakdown through various mechanisms. Tobacco smoke is rich in oxidants and reactive chemicals that can disrupt the body’s redox balance, impair neutrophil function, hinder fibroblast activity, alter blood vessel responses, and promote a destructive inflammatory phenotype [12,13]. In periodontal tissues, these effects may lead to a paradox: visible gingival inflammation and bleeding are often reduced due to smoking-induced vasoconstriction, even as deeper tissue destruction continues [14]. This highlights the importance of developing objective biological markers that may capture smoking-related inflammatory activity beyond traditional clinical assessments.

Neutrophils are the predominant leukocytes in the gingival crevice and play a crucial role in maintaining periodontal health. Under physiological conditions, they contribute to microbial containment through phagocytosis, degranulation, the production of reactive oxygen species, and the release of neutrophil extracellular traps (NETs) [15,16]. However, overactivation or dysregulation of neutrophils can lead to increased tissue damage and ongoing inflammation [17]. NETs are extracellular DNA-based structures decorated with histones, myeloperoxidase, neutrophil elastase, and other antimicrobial proteins [18]. Although NET formation enhances antimicrobial defense, persistent or excessive NETosis may promote collateral tissue injury, proteolytic degradation, oxidative damage, and inflammatory amplification [19,20]. Recent reviews highlight that NETs are increasingly recognized as active players in oral mucosal immunity and periodontal disease, but their specific role in human periodontitis remains incompletely understood [21,22].

Oxidative stress is an essential biological process that links smoking to periodontal tissue destruction. Excess reactive oxygen species can harm proteins, lipids, and nucleic acids, activate matrix metalloproteinases, and amplify inflammatory responses [23]. In periodontitis, oxidative stress closely correlates with connective tissue breakdown and bone loss [24]. Biomarkers in saliva and gingival crevicular fluid, such as 8-hydroxy-2′-deoxyguanosine, matrix metalloproteinase-8, interleukin-1β, interleukin-6, tumor necrosis factor-α, and total antioxidant capacity, have been suggested as non-invasive markers for periodontal inflammation and tissue damage [25]. Of these, MMP-8 and IL-1β are especially notable due to their links to periodontal disease detection and progression [26].

Although the association between smoking and periodontitis is well established, and recent studies have begun to describe smoking-associated NETosis-related and oxidative-inflammatory alterations in periodontal populations, several important gaps remain. Most prior studies compare current smokers with never-smokers only, leaving former smokers underrepresented despite their potential to reveal whether cessation is accompanied by partial biological normalization of the periodontal host response. Where NETosis-related biomarkers have been examined in smokers with periodontitis, they have generally been assessed as isolated markers rather than as part of a coordinated multi-pathway panel that simultaneously captures neutrophil activation, oxidative DNA damage, pro-inflammatory cytokine signaling, tissue degradation, and antioxidant capacity. Furthermore, biochemical verification of smoking status by salivary cotinine measurement in all participants—including never-smokers and former smokers—has not been consistently applied, leaving residual exposure misclassification as a concern in prior work. Finally, the strict exclusion of systemic diseases and interfering medications, necessary to isolate smoking-related biological effects from systemic inflammatory confounding, has not been uniformly implemented in existing studies. Consequently, a comprehensive, biochemically verified, three-group comparison of salivary NETosis-related and oxidative-inflammatory profiles in systemically healthy adults with periodontitis, supported by validated composite biomarker scores and robust sensitivity analyses, has not been previously reported.

This study aimed to compare salivary NETosis-related and oxidative-inflammatory biomarkers among current smokers, former smokers, and never-smokers with periodontitis and to examine their associations with periodontal severity. The main hypothesis was that current smokers would have higher salivary MPO-DNA complex levels and a higher combined NETosis score compared to former and never-smokers. Additional hypotheses suggested that smoking exposure might be dose-dependent, affecting NETosis and inflammatory biomarkers, and that these biomarkers could be associated with the extent of periodontal inflammation and tissue damage.

## 2. Materials and Methods

### 2.1. Study Design

This cross-sectional observational study was designed to evaluate the association between conventional-cigarette smoking status, salivary NETosis-related biomarkers, oxidative-inflammatory burden, and periodontal severity in systemically healthy adults with periodontitis. Participants were allocated into three parallel groups according to smoking status: current smokers, former smokers, and never-smokers. Each participant underwent a standardized full-mouth periodontal assessment, a structured smoking-exposure evaluation, and an unstimulated whole-saliva collection during a single study visit. The protocol was developed in accordance with the Declaration of Helsinki and followed the Strengthening the Reporting of Observational Studies in Epidemiology recommendations for observational research.

The study protocol was reviewed and approved by the Ethics Committee of Grigore T. Popa University of Medicine and Pharmacy, Iasi (approval number 5328/08.03.2018). All participants received verbal and written information regarding study objectives, clinical procedures, saliva collection, confidentiality, and data handling. Written informed consent was obtained before any study-related procedure.

All data were pseudonymized using unique study identification codes. Personal identifying information was stored separately from clinical and laboratory datasets and was accessible only to authorized research personnel.

### 2.2. Study Population

The study included 159 systemically healthy adult patients with periodontitis, equally distributed into three groups: current smokers with periodontitis (n = 53), former smokers with periodontitis (n = 53), and never-smokers with periodontitis (n = 53). Participants were recruited from the outpatient periodontal clinic of Grigore T. Popa University of Medicine and Pharmacy, Iasi. A purposive quota-sampling strategy was employed: patients attending the clinic for periodontal evaluation were screened consecutively against the pre-specified inclusion and exclusion criteria, and enrollment within each smoking-status group continued until the target of 53 participants per group was reached. This approach ensured balanced group allocation consistent with the a priori sample size calculation and prevented the numerical overrepresentation of any single smoking-status category. The complete participant flow, including the number of individuals screened, excluded with reasons, and enrolled per group, is presented in Supplementary Figure S1.

Participants were eligible if they were at least 18 years of age, had a diagnosis of periodontitis according to the 2017 World Workshop Classification of Periodontal and Peri-Implant Diseases and Conditions, had at least 20 natural teeth, had not received non-surgical or surgical periodontal treatment within the previous 6 months, and were able to provide written informed consent. In addition, only participants without known systemic diseases or chronic medication use that could influence inflammation, oxidative stress, neutrophil activity, salivary composition, vascular responses, or periodontal status were included.

Participants were excluded if they had diabetes mellitus, arterial hypertension, cardiovascular disease, autoimmune or autoinflammatory disease, chronic kidney disease, chronic liver disease, malignancy or history of malignancy, hematological disease, severe systemic inflammatory disease, acute systemic infection within the previous 4 weeks, or acute oral infection requiring emergency treatment. Further exclusion criteria were systemic antibiotic therapy within the previous 3 months, systemic corticosteroid therapy within the previous 3 months, immunosuppressive or immunomodulatory medication, long-term non-steroidal anti-inflammatory drug use, clinically relevant anticoagulant or antiplatelet therapy, antioxidant supplements or anti-inflammatory nutraceuticals within the previous 3 months, pregnancy or lactation, current orthodontic treatment, fewer than 20 natural teeth, periodontal therapy within the previous 6 months, oral mucosal ulcerative or inflammatory lesions unrelated to periodontitis, and current use of electronic cigarettes, heated tobacco products, smokeless tobacco, or nicotine replacement therapy.

### 2.3. Smoking Status Assessment

Smoking status was assessed using a structured questionnaire and supported by salivary cotinine measurement in all participants. Current smokers were defined as individuals who reported smoking at least one cigarette per day at the time of enrollment and regular smoking for at least the preceding 12 months. Former smokers were defined as individuals who had previously smoked regularly but had stopped smoking at least 12 months before enrollment. Never-smokers were defined as individuals who had smoked fewer than 100 cigarettes during their lifetime and did not currently use any tobacco or nicotine-containing product.

The questionnaire recorded cigarettes smoked per day, duration of smoking, age at smoking initiation, pack-years, time since cessation for former smokers, passive smoke exposure, and previous cessation attempts when applicable. Pack-years were calculated as cigarettes smoked per day divided by 20 and multiplied by years of smoking. 

Salivary cotinine concentration was measured in all participants using the Diagnostic Salivary Cotinine ELISA Kit (catalogue no. 1-2112; Salimetrics LLC, State College, PA, USA). The assay was performed according to the manufacturer’s instructions. Briefly, saliva samples, standards, and controls were analyzed in duplicate using the 96-well plate format, with 20 µL of saliva required per test. Absorbance was read at 450 nm using a calibrated microplate reader, and cotinine concentrations were calculated from the standard curve and expressed as ng/mL. According to the manufacturer, the assay has an analytical sensitivity of 0.15 ng/mL and a working range of 0.8–200 ng/mL. Of the 53 current smoker samples, 47 (88.7%) yielded cotinine concentrations exceeding the upper limit of quantification of 200 ng/mL (range of values requiring dilution: 227.0–442.5 ng/mL). These samples were diluted 1:4 in the manufacturer-supplied assay diluent and reanalyzed in duplicate. Final concentrations were back-calculated by multiplying the measured post-dilution value by the dilution factor of 4; all back-calculated results fell within the validated working range of the assay (post-dilution range: 56.8–110.6 ng/mL), confirming that all reported cotinine concentrations reflect values derived from within the validated quantitative range rather than extrapolations beyond the upper limit of detection. Dilution and reanalysis were performed strictly in accordance with the manufacturer’s validated protocol, which is designed to maintain assay linearity within the working range after appropriate sample dilution. Samples from former smokers (range: 1.0–23.8 ng/mL) and never-smokers were all within the assay working range and required no dilution.

Salivary cotinine was used as an objective continuous marker of recent nicotine exposure and as supportive biochemical information, but it was not used as the sole criterion for group allocation. 

### 2.4. Periodontal Clinical Examination

All participants underwent a comprehensive full-mouth periodontal examination performed by calibrated examiners. Measurements were recorded at six sites per tooth, namely mesiobuccal, buccal, distobuccal, mesiolingual or mesiopalatal, lingual or palatal, and distolingual or distopalatal sites. Third molars were excluded from periodontal charting.

Clinical examiners were not blinded to participants’ smoking status, as the smoking-exposure questionnaire, salivary cotinine collection, and periodontal examination were conducted during the same study visit. The potential for differential measurement bias was mitigated by the use of a standardized probe, a fixed six-site-per-tooth recording protocol, pre-study examiner calibration with acceptable intra- and inter-examiner agreement, and consensus-based resolution of disagreements regarding categorical periodontal classifications.

The recorded periodontal parameters were probing pocket depth, clinical attachment level, bleeding on probing, plaque index, gingival recession, number and percentage of sites with probing pocket depth of at least 4 mm, number and percentage of sites with probing pocket depth of at least 5 mm, number and percentage of bleeding sites, periodontal inflamed surface area, number of remaining teeth, periodontitis stage, and periodontitis grade. Probing pocket depth was defined as the distance from the gingival margin to the base of the periodontal pocket. Clinical attachment level was defined as the distance from the cemento-enamel junction to the base of the periodontal pocket. Bleeding on probing was recorded dichotomously as present or absent within 15 s after probing.

Clinical measurements were performed using a 15 UNC Color-Coded Periodontal Probe (PCPUNC15; HuFriedyGroup, Chicago, IL, USA). The same probe model was used throughout the study to ensure standardized probing of pocket depth and clinical attachment level measurements. Periodontitis staging and grading were assigned according to the 2017 World Workshop Classification of Periodontal and Peri-Implant Diseases and Conditions [4].

### 2.5. Examiner Calibration

Before study initiation, examiner calibration was performed on 10 adults with periodontitis who were not included in the final study sample. Two calibrated examiners independently recorded probing pocket depth and clinical attachment level at six sites per tooth—mesiobuccal, buccal, distobuccal, mesiolingual/palatal, lingual/palatal, and distolingual/palatal—excluding third molars. Each participant had at least 20 natural teeth, resulting in a minimum of 120 periodontal sites per participant and at least 1200 examined sites during the calibration procedure.

To assess intra-examiner reproducibility, each examiner repeated the measurements in the same participants after a 7-day interval, under the same clinical conditions, and using the same UNC-15 periodontal probe. To assess inter-examiner reproducibility, measurements obtained by the two examiners at the first calibration session were compared. Intra-examiner and inter-examiner agreement for probing pocket depth and clinical attachment level were evaluated using intraclass correlation coefficients based on a two-way random-effects model for absolute agreement.

Calibration was considered acceptable when intraclass correlation coefficients were ≥0.80 for both probing pocket depth and clinical attachment level. In the calibration phase, intra-examiner intraclass correlation coefficients were 0.91 and 0.89 for probing pocket depth and 0.90 and 0.88 for clinical attachment level, respectively, for the two examiners. Inter-examiner intraclass correlation coefficients were 0.86 for probing pocket depth and 0.84 for clinical attachment level. Bleeding on probing was recorded dichotomously, and inter-examiner agreement was assessed using Cohen’s kappa; a value of 0.82 was considered acceptable. Disagreements regarding categorical variables, including bleeding on probing and periodontitis staging, were resolved by discussion and consensus before study enrollment began.

### 2.6. Saliva Collection

Unstimulated whole saliva was selected as the biological matrix because it provides a non-invasive, repeatable, and clinically feasible method for assessing host-response biomarkers relevant to periodontal inflammation. Saliva was collected by passive drool into sterile polypropylene tubes. Participants were instructed to avoid eating, drinking except water, toothbrushing, mouth rinsing, chewing gum, and conventional cigarette smoking for at least 2 h before saliva collection. This 2 h abstinence period is consistent with standardized saliva collection protocols adopted in the periodontal and oral medicine literature and was intended to minimize confounding from immediate pre-analytical activities and the acute local effects of tobacco smoke on salivary composition. While this interval may not fully eliminate all residual acute effects of the most recently smoked cigarette on inflammatory and oxidative stress biomarkers, the graded biomarker pattern observed across current smokers, former smokers, and never-smokers—and the positive dose–response associations with cumulative pack-years—support the interpretation that the measured differences primarily reflect sustained, chronic exposure-related biological modulation rather than acute post-smoking responses.

Samples were collected between 08:00 and 11:00, before periodontal instrumentation, to reduce circadian variability and minimize contamination from procedure-induced bleeding.

Immediately after collection, samples were placed on ice and transported to the laboratory. Saliva was centrifuged at 3000× *g* for 15 min at 4 °C to remove cellular debris. The supernatant was transferred into sterile DNase/RNase-free polypropylene microcentrifuge tubes, aliquoted to avoid repeated thawing, and stored at −80 °C until biomarker analysis. Repeated freeze–thaw cycles were avoided. Samples visibly contaminated with blood were discarded and recollected, when possible, because blood contamination may interfere with salivary inflammatory, oxidative stress, and NETosis-related biomarker measurements.

### 2.7. Laboratory Processing and Biomarker Assessment

Laboratory personnel were blinded to participants’ smoking status and periodontal clinical data throughout biomarker analysis. The primary biomarker outcome was salivary myeloperoxidase-DNA complexes, used as an indicator of NETosis-related activity. MPO-DNA complexes were quantified using a human MPO-DNA complex ELISA kit (YPG0702; Elisabscience Biotechnology Co., Ltd., Wuhan, China), which recognizes native and recombinant human MPO-DNA complexes and reports intra- and inter-plate coefficients of variation below 10%. Citrullinated histone H3 was measured using the Citrullinated Histone H3 (Clone 11D3) ELISA Kit (catalogue no. 501620; Cayman Chemical, Ann Arbor, MI, USA), an assay designed to detect citrullinated histone H3 in human biological samples and relevant to NETosis assessment. Neutrophil elastase was quantified using the Human Neutrophil Elastase/ELA2 ELISA Kit (NBP1-91266; Novus Biologicals, Bio-Techne, Centennial, CO, USA), a colorimetric immunoassay for quantitative detection of human polymorphonuclear neutrophil elastase. Cell-free DNA was quantified using the Qubit™ dsDNA High Sensitivity Assay Kit (catalogue no. Q32851; Invitrogen™, Thermo Fisher Scientific, Waltham, MA, USA) on a Qubit™ 4 Fluorometer (catalogue no. Q33226; Invitrogen™, Thermo Fisher Scientific, Waltham, MA, USA), a fluorescence-based method for the selective quantification of double-stranded DNA (quantification range 0.1–120 ng). Samples and standards were mixed with the Qubit working solution, incubated at room temperature in the dark, and read using the dsDNA High Sensitivity assay setting. Results were expressed as ng/mL of saliva.

Oxidative-inflammatory biomarkers included matrix metalloproteinase-8, interleukin-1β, interleukin-6, tumor necrosis factor-α, 8-hydroxy-2′-deoxyguanosine, and total antioxidant capacity. Total MMP-8 was measured using the Human Total MMP-8 Quantikine ELISA Kit (DMP800B; R&D Systems, Bio-Techne, Minneapolis, MN, USA), a solid-phase ELISA designed to quantify total human MMP-8, including pro- and active forms, in biological matrices including saliva. IL-1β was quantified using the Human IL-1β/IL-1F2 Quantikine HS ELISA Kit (HSLB00D; R&D Systems, Bio-Techne, Minneapolis, MN, USA), which measures human IL-1β in serum, plasma, and saliva. IL-6 and TNF-α were measured using Human IL-6 Quantikine ELISA Kit (D6050; R&D Systems, Bio-Techne, Minneapolis, MN, USA) and Human TNF-alpha Quantikine ELISA Kit (DTA00D; R&D Systems, Bio-Techne, Minneapolis, MN, USA), respectively. Oxidative DNA damage was assessed using the DNA/RNA Oxidative Damage High Sensitivity ELISA Kit (catalogue no. 589320; Cayman Chemical, Ann Arbor, MI, USA), a competitive immunoassay that detects oxidized guanine species, including 8-OHdG. Total antioxidant capacity was determined using the Antioxidant Assay Kit (catalogue no. 709001; Cayman Chemical, Ann Arbor, MI, USA), a colorimetric assay based on the inhibition of ABTS oxidation to ABTS•+ by metmyoglobin; antioxidant capacity was quantified against a Trolox standard curve and expressed as Trolox equivalents (mmol/L). Because total antioxidant capacity reflects antioxidant defense rather than oxidative damage, values were reverse-coded when incorporated into the oxidative-inflammatory composite score, so that higher composite scores consistently represented greater oxidative-inflammatory burden.

Saliva processing was performed using laboratory equipment suitable for temperature-sensitive biological samples. Samples were centrifuged using a refrigerated benchtop centrifuge, Eppendorf Centrifuge 5702 R (Eppendorf SE, Hamburg, Germany), at 3000× *g* for 15 min at 4 °C. Aliquoting was performed using calibrated Eppendorf Research® plus single-channel and multichannel pipettes (Eppendorf SE, Hamburg, Germany). Absorbance for colorimetric ELISA assays was read using a Thermo Scientific™ Multiskan™ FC Microplate Photometer (Thermo Fisher Scientific, Waltham, MA, USA), equipped with a 450 nm filter and suitable for ELISA and other immunoassay applications. Samples were stored at −80 °C until analysis in a Thermo Scientific™ Forma™ 900 Series ultra-low-temperature freezer (Thermo Fisher Scientific, Waltham, MA, USA), designed for −50 °C to −86 °C sample storage.

All assays were performed according to the respective manufacturers’ instructions. Standards, blanks, internal controls, and saliva samples were analyzed in duplicate. Absorbance was read at the wavelength recommended by each assay protocol, typically 450 nm for colorimetric ELISA assays, and biomarker concentrations were calculated from standard curves generated according to the assay-specific instructions. Duplicate measurements with coefficients of variation greater than 15% were repeated. Biomarker concentrations below the lower limit of detection were handled according to a predefined rule by assigning half of the lower limit of detection. Values above the upper detection limit were remeasured after appropriate dilution.

### 2.8. Quality-Control Procedures

Pre-analytical and analytical variability were controlled through standardized participant preparation, morning sample collection whenever possible, immediate cooling of samples after collection, centrifugation under standardized conditions, aliquoting before storage, avoidance of repeated freeze–thaw cycles, duplicate measurement of all biomarkers, blinded laboratory analysis, reanalysis of samples with a duplicate coefficient of variation greater than 15%, and inclusion of assay-specific standards, blanks, and internal controls.

### 2.9. Composite Biomarker Scores

A composite NETosis score was calculated from MPO-DNA complexes, citrullinated histone H3, neutrophil elastase, and cell-free DNA. Because salivary biomarker distributions are commonly right-skewed, each component was log-transformed before standardization. Z-scores were computed using the overall sample mean and standard deviation (i.e., pooled across all three smoking-status groups), so that between-group differences in individual biomarkers were preserved in the composite index. The NETosis score was calculated as the arithmetic mean of the four standardized values. Higher scores indicated greater NETosis-related activity.

An oxidative-inflammatory composite score was calculated from MMP-8, IL-1β, IL-6, TNF-α, 8-OHdG, and total antioxidant capacity. Each biomarker was log-transformed where appropriate and standardized to z-scores using overall sample parameters. Total antioxidant capacity was reverse-coded before score construction. The oxidative-inflammatory score was calculated as the arithmetic mean of the six standardized values. Higher scores indicated greater oxidative-inflammatory burden.

Because composite scores aggregate multiple biomarkers that individually differ across smoking-status groups, their between-group effect sizes are expected to exceed those of any single constituent marker. The composite scores were constructed to capture coordinated pathway-level activity rather than to maximize group separation. Their clinical utility and discriminative performance require confirmation in independent cohorts.

The rationale for equal-weight averaging was threefold. First, assigning empirically derived weights from the same dataset would risk overfitting and reduce generalizability to independent cohorts. Second, equal weighting is transparent and reproducible across settings. Third, equal weights are appropriate when component biomarkers share comparable biological relevance within their respective pathways and when their standardized principal component loadings are similar in magnitude, as confirmed in the present data. Internal consistency of the composite scores was evaluated using Cronbach’s alpha applied to the log-transformed items. The NETosis composite yielded Cronbach’s α = 0.739, indicating acceptable internal consistency. The oxidative-inflammatory composite yielded Cronbach’s α = 0.679, which is borderline but expected given that this score intentionally aggregates markers from distinct yet interrelated biological processes—collagen degradation (MMP-8), pro-inflammatory cytokine signaling (IL-1β, IL-6, TNF-α), oxidative DNA damage (8-OHdG), and antioxidant defense (TAC)—rather than replicate indicators of a single latent trait. Principal component analysis on the log-transformed items confirmed a dominant first component for both composites, explaining 57.0% of variance for the NETosis score and 42.5% for the oxidative-inflammatory score, with near-equal loadings across all component markers (NETosis PC1 loadings: 0.487–0.511; oxidative-inflammatory PC1 loadings: 0.364–0.485), supporting the use of an unweighted average. Spearman inter-item correlations were moderate and uniformly positive within each composite (NETosis items: ρ = 0.374–0.462; oxidative-inflammatory items: ρ = 0.126–0.490). Cross-composite Spearman correlations between all ten log-transformed biomarkers ranged from 0.126 to 0.517, indicating that the two scores captured related but non-redundant aspects of the smoking-associated biological response. The complete inter-item Spearman correlation matrix is provided in Supplementary Table S4.

### 2.10. Covariates

The collected covariates were age, sex, body mass index, plaque index, number of remaining teeth, periodontitis stage, periodontitis grade, oral hygiene habits, frequency of dental visits, and alcohol consumption. Body mass index was calculated as body weight (kg) divided by height (m) squared. Because systemic diseases and interfering chronic medications were excluded by design, diabetes, arterial hypertension, cardiovascular disease, autoimmune disease, renal disease, hepatic disease, malignancy, and chronic medication use were not included as adjustment covariates.

### 2.11. Sample Size Calculation

The sample size was calculated to detect differences in salivary MPO-DNA complex levels among the three smoking-status groups using a one-way analysis of variance framework. Based on an anticipated effect size of Cohen’s *f* = 0.25 (small-to-medium), a two-sided significance level of α = 0.05, statistical power of 80%, three groups, and equal allocation, G*Power (version 3.1.9.7; Heinrich-Heine-Universität Düsseldorf, Düsseldorf, Germany) indicated a required total sample size of 159 participants. Therefore, 53 participants were enrolled per group. The assumed effect size was selected conservatively based on previously reported differences in salivary inflammatory biomarkers between smokers and non-smokers with periodontitis. The observed effect size for the primary outcome (*η*^2^ = 0.317, corresponding to Cohen’s *f* = 0.68) exceeded the a priori assumption, indicating that the study was adequately powered for the primary comparison.

### 2.12. Statistical Analysis

All statistical analyses were performed in R (version 4.3.2, R Foundation for Statistical Computing, Vienna, Austria). Continuous variables were inspected for distribution using histograms, Q–Q plots, and the Shapiro–Wilk test applied within each smoking-status group. Homogeneity of variance was assessed using Levene’s test. Normally distributed variables were reported as mean and standard deviation, while non-normally distributed variables were reported as median and interquartile range. Categorical variables were presented as numbers and percentages.

Baseline demographic and clinical characteristics were compared among current smokers, former smokers, and never-smokers using one-way analysis of variance for normally distributed continuous variables, the Kruskal–Wallis test for non-normally distributed variables, and the chi-square test or Fisher’s exact test for categorical variables.

The primary analysis compared salivary MPO-DNA complex levels among the three smoking-status groups. Salivary MPO-DNA complexes showed significant departure from normality in all three groups (Shapiro–Wilk *p* < 0.05 in each); however, log-transformation achieved normality. Because one-way analysis of variance is robust to moderate departures from normality at equal sample sizes (n = 53 per group), the primary between-group comparison was conducted on both untransformed and log-transformed values. Additionally, the non-parametric Kruskal–Wallis test was applied as a sensitivity check; all three approaches yielded concordant results. Post hoc pairwise comparisons were performed using Tukey’s honestly significant difference test. Welch’s analysis of variance was additionally computed to account for potential variance heterogeneity. Distributional assessments and sensitivity analyses for all individual biomarkers are reported in Supplementary Table S1.

To address multiplicity, a pre-specified analytical hierarchy was adopted. The primary outcome (salivary MPO-DNA complexes) was tested at the conventional two-sided significance level of α = 0.05 without adjustment for multiple comparisons. Secondary biomarker analyses (citrullinated histone H3, neutrophil elastase, cell-free DNA, MMP-8, IL-1β, IL-6, TNF-α, 8-OHdG, total antioxidant capacity, composite NETosis score, and oxidative-inflammatory score; 11 tests) were corrected for multiplicity using the Benjamini–Hochberg false discovery rate (FDR) procedure applied across the 11 omnibus one-way ANOVAs. Dose–response correlation analyses (Spearman coefficients between three smoking-exposure variables and six biomarker/periodontal-severity outcomes; 18 tests) were similarly corrected using the Benjamini–Hochberg procedure. Interaction analyses between smoking status and NETosis-related biomarkers on periodontal severity outcomes were pre-specified as exploratory, and their *p*-values are reported without adjustment for multiplicity.

Adjusted analyses were performed using multivariable linear regression. The primary adjusted model used log-transformed MPO-DNA complexes as the dependent variable and smoking status as the main independent variable, adjusted for age, sex, body mass index, plaque index, number of remaining teeth, periodontitis stage, and periodontitis grade.

As a sensitivity analysis, between-group comparisons for the primary and secondary biomarker outcomes were repeated in the subgroup of participants with Stage III periodontitis (n = 126) to evaluate whether findings were robust after restricting the analysis to a more homogeneous disease-severity stratum. An additional sensitivity analysis excluded former smokers with salivary cotinine concentrations exceeding 10 ng/mL (n = 24 excluded) to evaluate whether findings were robust when restricting the former-smoker group to individuals with minimal residual nicotine exposure.

Associations between smoking exposure and biomarker concentrations were evaluated using Spearman correlation coefficients. Smoking exposure variables included cigarettes per day, pack-years, smoking duration, salivary cotinine concentration, and exhaled carbon monoxide. Additional adjusted linear regression models evaluated whether smoking exposure remained associated with biomarker levels after controlling for age, sex, body mass index, plaque index, and number of remaining teeth.

Associations between biomarkers and periodontal severity were evaluated using correlation and multivariable regression analyses. Periodontal severity outcomes included mean probing pocket depth, mean clinical attachment level, percentage of bleeding on probing, number of sites with probing pocket depth ≥ 5 mm, and periodontal inflamed surface area. Regression models were adjusted for smoking status, age, sex, body mass index, plaque index, number of remaining teeth, periodontitis stage, and periodontitis grade.

Exploratory interaction analyses examined whether associations between NETosis-related biomarkers and periodontal severity differed by smoking status. Interaction terms between smoking group and MPO-DNA complexes (or the composite NETosis score) were included in adjusted regression models. These analyses were considered hypothesis-generating, and *p*-values are reported without correction for multiplicity.

All statistical tests were two-sided. Statistical significance was defined as *p* < 0.05 for the primary outcome and FDR-adjusted *p* < 0.05 for secondary biomarker comparisons.

### 2.13. Data Management

Data were entered into a password-protected electronic database and checked for accuracy before statistical analysis. Biological samples and laboratory results were labeled only with participant study codes. The final analytical dataset contained no directly identifying information. No missing values were present in the final dataset; therefore, complete-case analysis was performed.

## 3. Results

### 3.1. Demographic and Periodontal Characteristics

The final analysis included 159 participants with periodontitis, equally distributed across the three smoking-status groups: current smokers (n = 53), former smokers (n = 53), and never-smokers (n = 53).

Demographic and clinical characteristics are summarized in Table 1. Sex distribution and body mass index were comparable across groups. Former smokers were significantly older than never-smokers (mean 52.3 ± 8.8 vs. 47.5 ± 9.7 years; Tukey *p* < 0.05), while current smokers (48.5 ± 5.9 years) did not differ significantly from either group (*F* = 4.98, *p* = 0.008, *η*^2^ = 0.060); age was therefore included as a covariate in all multivariable models.

Current smokers exhibited significantly higher plaque index, deeper mean PPD, greater mean CAL, more sites with PPD ≥ 5 mm, and greater PISA than both former and never-smokers, who in turn differed significantly from each other for mean PPD, mean CAL, sites with PPD ≥ 5 mm, and PISA (Table 1).

Bleeding on probing was higher in current smokers (52.1 ± 9.3%) and former smokers (50.9 ± 7.8%) than in never-smokers (46.8 ± 8.9%) in unadjusted comparisons. However, this difference was not significant after adjustment for probing pocket depth and plaque index (*p* = 0.34), consistent with the higher disease severity observed among current smokers rather than an independent effect of smoking on gingival bleeding.

Periodontitis stage and grade differed significantly according to smoking status (Table 2). Stage III periodontitis was more frequent among current smokers than among former and never-smokers, being present in 96.2% of current smokers, 86.8% of former smokers, and 54.7% of never-smokers (*p* < 0.001). Similarly, Grade C periodontitis was markedly more prevalent among current smokers, affecting 88.7% of current smokers compared with 24.5% of former smokers and 20.8% of never-smokers (*p* < 0.001). Because periodontitis stage and grade differed across smoking groups, both variables were included in adjusted models to reduce confounding by disease severity.

### 3.2. Smoking Exposure and Biochemical Validation

Smoking exposure variables are reported in Table 3. Current smokers had a mean cigarette consumption of 15.02 ± 4.97 cigarettes/day and a mean cumulative exposure of 22.54 ± 10.30 pack-years. Former smokers had a lower cumulative exposure (13.49 ± 7.23 pack-years) and a mean cessation interval of 8.39 ± 4.12 years.

Salivary cotinine concentrations confirmed clear biochemical separation between groups: current smokers (312.3 ± 77.0 ng/mL), former smokers (9.7 ± 5.1 ng/mL), and never-smokers (3.2 ± 1.4 ng/mL). The lowest cotinine value among current smokers (132.6 ng/mL) exceeded the highest value among former smokers (23.8 ng/mL) by more than 100 ng/mL, confirming that no misclassification of active smoking status occurred. 

**Figure 1:**
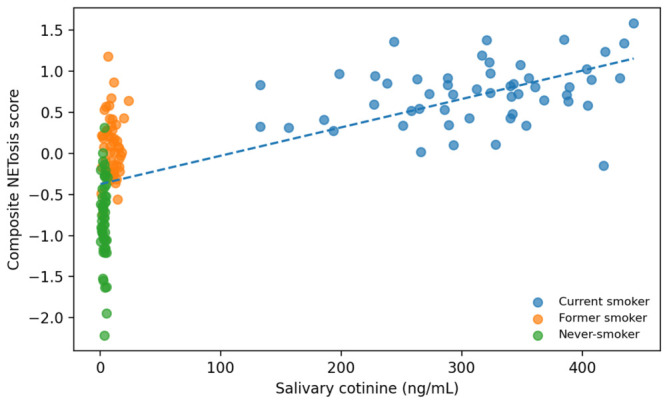
Association between salivary cotinine and composite NETosis score. Each point represents one participant; the dashed line represents the overall linear trend.

Among former smokers, detectable cotinine levels (range 1.0–23.8 ng/mL) were consistent with passive environmental tobacco smoke exposure rather than active use. Exhaled carbon monoxide showed the same group pattern. The distribution of salivary cotinine and its relation to NETosis activity are illustrated in Figure 1.

### 3.3. Salivary NETosis-Related Biomarkers

The primary biomarker outcome, salivary MPO-DNA complexes, differed significantly among the three smoking-status groups (*F* = 36.22, *p* <0.001, *η*^2^ = 0.317; Table 4). Because MPO-DNA complexes showed significant departure from normality in all three groups (Shapiro–Wilk: current smokers *p* = 0.025, former smokers *p* = 0.001, never-smokers *p* < 0.001), analyses were confirmed using log-transformed one-way analysis of variance (*F* = 45.89, *p* < 0.001, *η*^2^ = 0.370) and the Kruskal–Wallis test (*H* = 58.32, *p* < 0.001), both yielding concordant results. 

Log-transformation restored normality in all groups (Shapiro–Wilk *p* > 0.25 for all groups). Raw descriptive values are reported in Table 4 for clinical interpretability. Mean MPO-DNA levels were highest in current smokers, intermediate in former smokers, and lowest in never-smokers.

**Figure 2:**
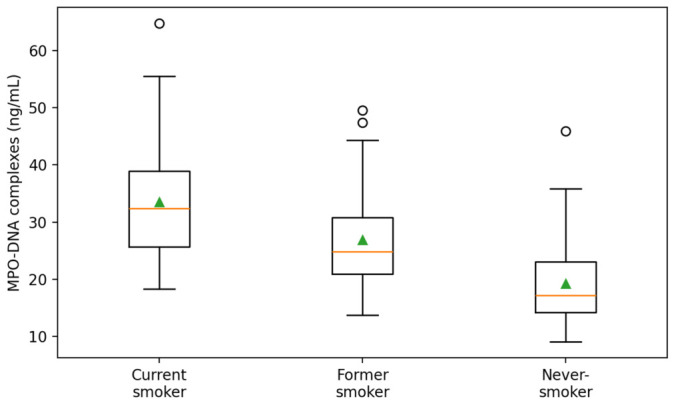
Salivary MPO-DNA complex concentrations according to smoking status. Boxes represent interquartile ranges, horizontal lines indicate medians, and markers indicate group means.

Tukey post hoc testing revealed significant differences for all pairwise comparisons (Table 5). The distribution of MPO-DNA complexes across smoking groups is presented in Figure 2. Concordance between raw ANOVA, log-transformed ANOVA, and Kruskal–Wallis testing was confirmed for all individual biomarkers (Supplementary Table S1).

### 3.4. Oxidative-Inflammatory Biomarkers

Secondary biomarker analyses demonstrated a consistent smoking-associated biological gradient (Table 4). Citrullinated histone H3, neutrophil elastase, cell-free DNA, MMP-8, IL-1β, IL-6, TNF-α, and 8-OHdG were significantly higher in current smokers than in former and never-smokers after false-discovery-rate correction. Conversely, total antioxidant capacity was lowest in current smokers and highest in never-smokers. All secondary biomarker comparisons remained statistically significant after Benjamini–Hochberg FDR correction (adjusted *p*-values ranged from 1.55 × 10^−42^ to 1.05 × 10^−8^).

**Figure 3:**
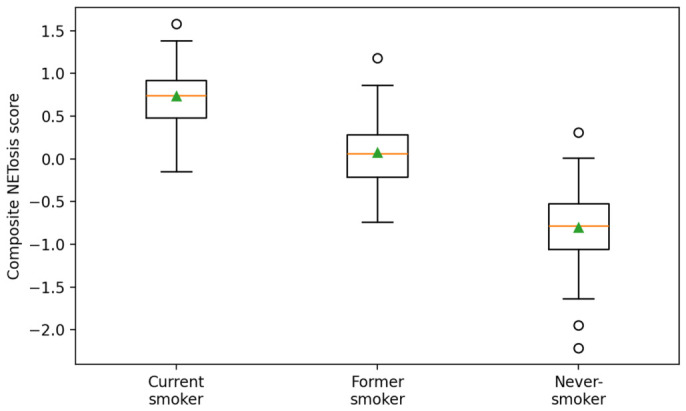
Composite NETosis score according to smoking status.

**Figure 4:**
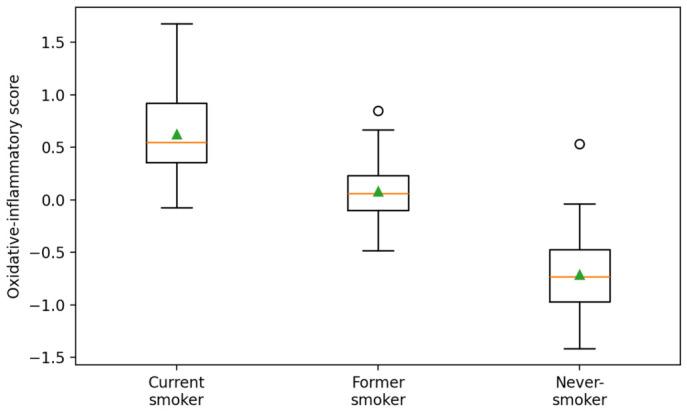
Oxidative-inflammatory score according to smoking status.

The composite NETosis score differed strongly among groups (*F* = 183.46, *p* < 0.001, *η*^2^ = 0.702), as did the oxidative-inflammatory score (*F* = 198.36, *p* < 0.001, *η*^2^ = 0.718). Figure 3 and Figure 4 show the distribution of the composite NETosis and oxidative-inflammatory scores, respectively.

### 3.5. Dose–Response Associations

Among participants with current or former smoking exposure, pack-years, salivary cotinine, and exhaled carbon monoxide were positively associated with NETosis and oxidative-inflammatory markers (Table 6). All 18 exposure–biomarker and exposure–severity correlations remained statistically significant after Benjamini–Hochberg FDR correction (adjusted *p*-values ranged from 4.91 × 10^−12^ to 0.018).

**Figure 5:**
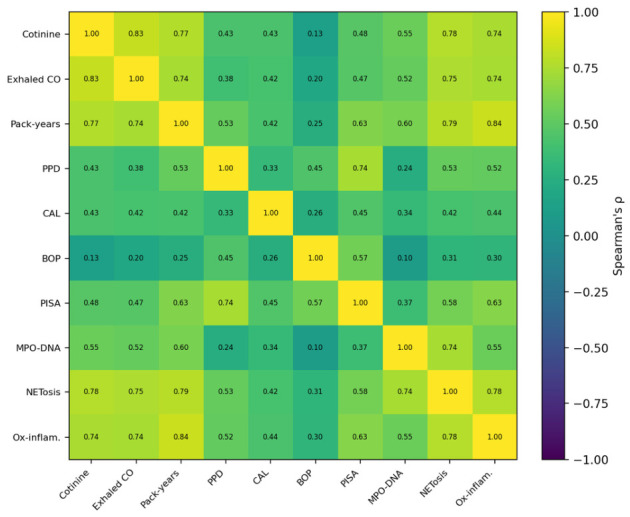
Spearman correlation matrix of smoking exposure, periodontal severity, and salivary biomarker variables.

Pack-years showed moderate correlations with the composite NETosis score, oxidative-inflammatory score, and periodontal inflamed surface area. Salivary cotinine was strongly associated with the composite NETosis score and oxidative-inflammatory score, supporting a dose–response relationship between biochemical tobacco exposure and the salivary host-response phenotype. The global correlation structure among smoking exposure, clinical severity, and biomarker variables is displayed in Figure 5.

### 3.6. Multivariable Regression Analyses

In adjusted linear regression models, smoking status remained independently associated with the primary NETosis biomarker and the composite NETosis score after controlling for age, sex, body mass index, plaque index, number of remaining teeth, periodontitis stage, and periodontitis grade (Table 7). Compared with never-smokers, both former smokers and current smokers had higher log-transformed MPO-DNA levels, with the largest adjusted effect observed in current smokers. A similar pattern was observed for the composite NETosis score. In models using periodontal inflamed surface area as the outcome, the oxidative-inflammatory score and plaque index remained independently associated with inflammatory periodontal burden, whereas the independent association for the NETosis score was attenuated after simultaneous adjustment for smoking group and oxidative-inflammatory activity.

### 3.7. Interaction Analyses

Exploratory interaction analyses examined whether the associations between NETosis-related biomarkers and periodontal severity differed by smoking status. Interaction terms between smoking group and MPO-DNA complexes, and between smoking group and the composite NETosis score, were included in adjusted regression models with PISA, mean PPD, mean CAL, BOP, and the number of sites with PPD ≥ 5 mm as outcomes. 

None of the interaction terms reached statistical significance (all *p* > 0.10), and the addition of interaction terms did not meaningfully improve model fit (ΔR^2^ < 0.011 for all models). These findings suggest that the associations between salivary NETosis biomarkers and periodontal severity were consistent across smoking-status groups. These interaction analyses were pre-specified as exploratory. *p*-values are reported without adjustment for multiplicity and should be interpreted as hypothesis-generating.

### 3.8. Sensitivity Analysis

In the sensitivity analysis restricted to participants with Stage III periodontitis (n = 126; current smokers n = 51, former smokers n = 46, never-smokers n = 29), all primary and secondary biomarker differences remained statistically significant. MPO-DNA complexes showed the same graded pattern (current smokers 33.67 ± 10.12, former smokers 26.86 ± 8.32, never-smokers 20.94 ± 8.18 ng/mL; *F* = 19.09, *p* < 0.001, *η*^2^ = 0.237), as did the composite NETosis score (*F* = 127.65, *p* < 0.001, *η*^2^ = 0.675) and the oxidative-inflammatory score (*F* = 137.23, *p* < 0.001, *η*^2^ = 0.691). 

All individual secondary biomarkers remained significant in this subgroup (all *p* < 0.001). These findings indicate that between-group biomarker differences were not attributable to the unequal distribution of periodontitis stage across smoking-status groups (Supplementary Table S2).

In a sensitivity analysis excluding former smokers with salivary cotinine > 10 ng/mL (retaining n = 29 former smokers with mean cotinine 6.0 ng/mL), all between-group differences remained significant for MPO-DNA complexes (*F* = 36.10, *p* < 0.001) and the composite NETosis score (*F* = 172.39, *p* < 0.001), confirming that findings were not driven by potentially misclassified former smokers (Supplementary Table S3).

## 4. Discussion

The present study identified a distinct salivary host-response profile in current smokers with periodontitis, characterized by higher NETosis-related activity and a greater oxidative-inflammatory burden compared with former and never-smokers. In this systemically healthy cohort, from which diabetes mellitus, arterial hypertension, cardiovascular disease, autoimmune conditions, chronic kidney or liver disease, malignancy, and potentially interfering chronic medication were excluded, the observed differences are more plausibly consistent with smoking-associated biological modulation rather than major systemic inflammatory confounding. The main findings support the concept that cigarette smoking is not only associated with more severe periodontal clinical expression but also with a measurable salivary signature of markers consistent with neutrophil activation, oxidative stress, and inflammatory tissue degradation. Because periodontitis stage and grade differed across smoking groups, these variables were included in adjusted models, and smoking status remained independently associated with MPO-DNA complexes and the NETosis score.

A central observation was the elevation of salivary MPO-DNA complexes in current smokers. MPO-DNA complexes are widely interpreted as a biologically relevant indicator of neutrophil extracellular trap formation, because they reflect the association between extracellular DNA and myeloperoxidase, a neutrophil granule enzyme released during NET formation [27]. The concurrent increase in citrullinated histone H3, neutrophil elastase, cell-free DNA, and the composite NETosis score suggests that the findings do not reflect an isolated analytical fluctuation but rather a coordinated neutrophil-derived biological pattern. This interpretation is consistent with recent reviews emphasizing that neutrophils and NETs have dual roles in oral mucosal defense and periodontal inflammation, acting protectively under controlled conditions but contributing to tissue injury when activation is excessive or unresolved [19,28].

The higher NETosis-related biomarker profile observed among current smokers is biologically plausible. Cigarette smoke contains abundant oxidants and reactive chemical compounds that can alter neutrophil function, increase oxidative burst activity, disturb redox homeostasis, and intensify inflammatory signaling [29]. In periodontal tissues, smoking-related priming may shift the host response toward a more destructive phenotype [30]. Rather than simply reflecting bacterial challenge, the salivary NETosis-related biomarker profile may therefore represent a host-derived imprint of tobacco exposure on periodontal inflammation. Recent clinical evidence has reported elevated salivary NETosis-related biomarkers in smokers with periodontitis, supporting the relevance of this pathway in human periodontal disease [31].

An original aspect of the present study is the inclusion of former smokers as a separate group. Former smokers generally showed intermediate biomarker values between current smokers and never-smokers, suggesting that smoking cessation may be accompanied by partial biological normalization of the periodontal inflammatory environment. This does not prove reversibility, because the study was cross-sectional; nevertheless, the graded pattern across current, former, and never-smokers is informative. It suggests that the salivary NETosis-related and oxidative-inflammatory profile may retain an association with previous exposure while being most pronounced under active smoking conditions. This finding supports the clinical value of distinguishing former smokers from never-smokers rather than merging both categories into a single non-smoking reference group.

Furthermore, current smokers exhibited higher levels of markers related to tissue degradation, inflammatory signaling, and oxidative damage, including MMP-8, IL-1β, IL-6, TNF-α, and 8-OHdG, together with an unfavorable antioxidant profile. MMP-8 is especially relevant in periodontitis because it participates directly in collagen breakdown and connective tissue destruction [32], while IL-1β is a key mediator of periodontal inflammatory amplification [33]. Recent salivary biomarker studies and systematic reviews continue to support the diagnostic and staging relevance of IL-1β and MMP-8 in periodontal disease, while also emphasizing that these molecules are best interpreted as part of a broader inflammatory profile rather than as standalone disease-specific markers [26,34].

The positive associations between smoking exposure indices and biomarker concentrations provide an additional layer of understanding. Pack-years, cigarettes per day, and salivary cotinine were associated with NETosis and oxidative-inflammatory markers, indicating that the host-response phenotype was not only related to categorical smoking status but also to the intensity of tobacco exposure. The detectable cotinine concentrations observed in former smokers require cautious interpretation. These values were markedly lower than those recorded in current smokers and may reflect passive exposure to environmental tobacco smoke, individual differences in nicotine metabolism, or occasional unreported exposure. For this reason, cotinine was treated as a continuous marker of recent nicotine exposure and supportive biochemical validation rather than as the sole determinant of smoking-status classification. Importantly, the large separation between current smokers and the other groups supports the validity of the primary exposure contrast. Furthermore, a sensitivity analysis excluding former smokers with cotinine concentrations above 10 ng/mL yielded unchanged results, providing additional assurance that the intermediate biomarker profile of former smokers reflects genuine biological attenuation rather than residual active smoking.

The association between salivary biomarkers and periodontal severity is clinically meaningful. Higher NETosis and oxidative-inflammatory scores were associated with deeper probing pocket depths, greater clinical attachment loss, a higher bleeding burden, an increased number of deep periodontal sites, and a greater periodontal inflamed surface area. These findings suggest that the biomarker profile was associated not only with tobacco exposure but also with the intensity of periodontal inflammatory destruction. Importantly, the persistence of these associations after adjustment for relevant covariates suggests that NETosis-related biomarker levels are associated with periodontal inflammatory burden independently of common clinical covariates, though whether they provide clinically meaningful complementary information beyond conventional periodontal charting requires prospective validation. Notably, although unadjusted bleeding on probing appeared higher in current smokers, this difference was attributable to greater pocket depth and plaque accumulation rather than to a direct smoking effect on gingival vascularity. When disease severity was accounted for, current smokers showed a tendency toward lower bleeding per unit of periodontal destruction, consistent with the well-documented vasoconstrictive effect of tobacco. This observation reinforces the rationale for salivary biomarker-based assessment, because clinical bleeding indices may not fully reflect the inflammatory burden in smokers.

The results also fit with contemporary evidence that smoking influences both periodontal inflammation and tissue destruction. Meta-analytic data have reinforced the association between smoking, periodontal inflammation, and alveolar bone resorption, while also highlighting the complexity of biomarker interpretation across heterogeneous populations [11]. Earlier evidence established smoking as a major determinant of periodontitis incidence and progression, but recent research increasingly focuses on the biological phenotype induced by smoking rather than on epidemiological association alone [35,36]. The present study contributes to this direction by proposing a NETosis-related and oxidative-inflammatory phenotype as one possible biological expression of smoking-associated periodontitis.

From a translational perspective, saliva is an accessible biological matrix for host-response profiling, being non-invasive, repeatable, and compatible with standardized collection protocols. In smoking-associated periodontitis, salivary biomarker assessment may be of particular interest given that tobacco-related vascular effects may not fully reflect the underlying inflammatory burden through conventional clinical indices such as bleeding on probing. However, the present study did not include diagnostic performance analyses, receiver operating characteristic curve analyses, or predictive validation, and no comparative claim regarding the clinical superiority of the biomarker panel over conventional periodontal assessment can be made on the basis of the current data alone. Whether a panel including MPO-DNA complexes, citrullinated histone H3, neutrophil elastase, MMP-8, IL-1β, 8-OHdG, and total antioxidant capacity would offer clinically meaningful discriminative or predictive value beyond standard periodontal charting remains to be established in prospective, adequately powered validation studies.

The composite NETosis and oxidative-inflammatory scores are another strength of the analytical approach. Individual biomarkers are vulnerable to biological variability, pre-analytical influences, and assay-specific noise. Composite scores can reduce this instability by capturing pathway-level activity rather than isolated molecular changes. In the present study, the composite scores strengthened the biological narrative by showing coordinated activation of neutrophil-derived and oxidative-inflammatory pathways. This approach may be particularly useful in periodontology, where tissue destruction results from interactions among microbial, immune, oxidative, and behavioral factors rather than a single dominant mediator [37]. A methodological consideration is that the large effect sizes observed for the composite scores (*η*^2^ = 0.702 and 0.718) partly reflect the aggregation of multiple smoking-sensitive biomarkers into a single index. This amplification is a mathematical property of averaging correlated z-scores that individually differ between groups and does not imply circularity, because the constituent biomarkers were selected based on prior biological evidence linking NETosis and oxidative stress to periodontal inflammation, not on their observed group differences in the present sample. Notably, the individual constituent biomarkers already showed large effect sizes prior to aggregation, ranging from *η*^2^ = 0.210 for IL-1β to *η*^2^ = 0.566 for total antioxidant capacity, indicating that the amplification observed in the composite scores reflects a genuine and strong underlying biological gradient rather than a statistical artifact of the averaging procedure.

Internal consistency analysis (Cronbach’s *α* = 0.739 for the NETosis score and *α* = 0.679 for the oxidative-inflammatory score) and principal component analysis (PC1 explaining 57.0% and 42.5% of variance, respectively, with near-equal loadings across all component markers) supported the use of an unweighted averaging approach and confirmed that each composite captured a dominant shared dimension among its constituent biomarkers. The moderate inter-item and cross-composite correlations (*ρ* = 0.126–0.517) further indicated that the two scores reflect related but non-redundant aspects of the smoking-associated biological response. Nevertheless, the composite scores were derived and evaluated in the same cohort, and external validation in independent populations is needed before their clinical applicability can be established.

The exclusion of major systemic diseases and potentially interfering medication represents a deliberate methodological choice. Diabetes mellitus, arterial hypertension, cardiovascular disease, autoimmune disease, renal or hepatic disease, malignancy, corticosteroids, immunosuppressants, long-term anti-inflammatory medication, and antihypertensive drugs can all influence oxidative stress, neutrophil behavior, inflammatory cytokines, salivary composition, or periodontal vascular responses [38,39,40,41]. By excluding these factors, the present study prioritized internal validity and biological interpretability. This stricter design, however, also narrows external generalizability. In real-world periodontal populations, smoking frequently coexists with systemic conditions and medication use. 

Moreover, a sensitivity analysis restricted to Stage III periodontitis yielded concordant results across all biomarker outcomes, indicating that the observed smoking-associated differences are not explained by the higher prevalence of advanced disease among current smokers. This subgroup analysis, together with the covariate-adjusted regression models, supports the interpretation that smoking status contributes independently to the salivary NETosis-related and oxidative-inflammatory profile beyond its association with periodontal disease severity.

A further interpretive aspect concerns residual confounding by periodontal inflammatory burden. Although smoking status remained independently associated with MPO-DNA complexes and the composite NETosis score after adjusting for periodontitis stage and grade, and although the Stage III sensitivity analysis yielded concordant results, complete separation of smoking-specific biological effects from those attributable to greater disease severity cannot be guaranteed. Periodontitis stage and grade are categorical constructs; within each category, continuous variation in probing pocket depth, clinical attachment level, and periodontal inflamed surface area was still evident between smoking-status groups (Table 1). Current smokers showed higher mean PPD, CAL, and PISA than former and never-smokers even within the Stage III subgroup, and the biomarker differences may therefore partly reflect a greater local inflammatory and tissue-destructive burden rather than a direct effect of tobacco exposure on neutrophil activation and oxidative stress. Future studies employing continuous disease-severity indices as covariates, or designs that match participants on periodontal severity before comparing smoking groups, would provide stronger evidence for the independent contribution of smoking to the salivary host-response phenotype observed here.

The study has limitations. Its cross-sectional design prevents causal inference. Although smoking status and cotinine levels were associated with NETosis and oxidative-inflammatory biomarkers, causal relationships between smoking exposure, NETosis-related activity, and periodontal destruction cannot be inferred.

A further limitation is the potential for residual confounding by periodontal disease severity. Although smoking status remained independently associated with the primary and composite biomarker outcomes after adjustment for periodontitis stage and grade, and although the Stage III sensitivity analysis yielded concordant results, these measures may not fully account for continuous variation in clinical severity within each disease category. Current smokers retained substantially higher mean probing pocket depth, clinical attachment level, and periodontal inflamed surface area than former and never-smokers even within the Stage III subgroup, and the observed biomarker elevations may therefore partly reflect a greater local inflammatory and tissue-destructive burden rather than an exclusive effect of tobacco exposure on neutrophil activation and oxidative stress pathways. Complete separation of smoking-specific biological effects from those attributable to greater disease severity cannot be guaranteed within the constraints of the present design.

Additionally, clinical periodontal examiners were not blinded to participants’ smoking status, as the smoking-exposure assessment and clinical examination were conducted during the same visit, and complete blinding was not feasible. Although the standardized measurement protocol, calibrated examiners, and objective probe-dependent measurements limit the scope for examiner-introduced bias, the possibility that knowledge of smoking status subtly influenced clinical recording or staging decisions cannot be entirely excluded.

Saliva provides a whole-mouth and partially systemic signal rather than a site-specific measurement. Salivary biomarker concentrations represent the integrated output of all periodontal sites, major and minor salivary gland secretions, oral mucosal surfaces, and supragingival plaque-associated microbial products, and cannot be attributed to individual periodontal pockets or to a single biological source. A particularly relevant consideration in the present study is the contribution of gingival crevicular fluid to the total salivary biomarker pool. GCF volume is proportional to the degree of local periodontal inflammation and pocket depth; because current smokers had significantly deeper pockets and greater periodontal inflamed surface area than the other groups, their higher salivary biomarker concentrations may partly reflect a greater GCF contribution to the salivary matrix rather than exclusively reflecting smoking-associated neutrophil priming or oxidative stress. Although this effect cannot be fully eliminated in a whole-saliva design, it is analogous to the residual confounding concern discussed above and represents an inherent constraint of non-invasive salivary profiling in populations with unequal disease severity. Individual differences in unstimulated salivary flow rate represent a further source of variability, as lower flow rates concentrate soluble biomarkers independently of their biological production. Although saliva was collected under standardized fasting and resting conditions in the morning to minimize circadian and procedural variability, salivary flow rate was not measured as a covariate in the present study, and its potential confounding influence cannot be excluded. Additionally, while participants with oral mucosal lesions unrelated to periodontitis were excluded, other oral factors such as caries activity and supragingival plaque accumulation—which differed significantly across groups—may also contribute to the salivary inflammatory milieu. Gingival crevicular fluid or tissue-based analyses in future studies could provide more localized and source-specific information on the periodontal pocket biology underlying the smoking-associated phenotype observed here.

Moreover, although a standardized 2 h pre-collection abstinence period was applied, this interval may not fully eliminate all acute physiological effects of the most recent cigarette on salivary oxidative stress and inflammatory biomarker concentrations in current smokers. Future studies employing longer standardized abstinence periods—such as overnight abstinence verified by exhaled carbon monoxide measurement immediately before collection—would more effectively separate acute from chronic smoking-related effects on the salivary host-response phenotype.

A further consideration is that salivary biomarkers may reflect systemic as well as local oral sources. Pro-inflammatory cytokines, including IL-6, TNF-α, and IL-1β, can enter saliva from the systemic circulation via ultrafiltration across salivary gland epithelium or via GCF as an inflammatory exudate, and oxidative stress markers such as 8-OHdG may capture systemic oxidative damage in addition to local periodontal tissue injury. Although the exclusion of systemic diseases and interfering medications was designed to minimize systemic inflammatory confounding, subclinical systemic differences among smoking-status groups that fall below clinical diagnostic thresholds cannot be entirely excluded and may have contributed to the observed biomarker gradient independently of periodontal NETosis-related activity.

A related limitation concerns the individual specificity of the NETosis-related markers included in the panel. MPO-DNA complexes and citrullinated histone H3 are considered relatively specific indicators of NET formation—reflecting, respectively, the extracellular co-localization of DNA with a neutrophil-specific granule enzyme and PAD4-mediated chromatin decondensation during NETosis. By contrast, neutrophil elastase is stored in azurophilic granules and is also released during classical degranulation independently of NET formation; elevated salivary levels may therefore reflect broader neutrophil activation rather than NETosis exclusively. It should further be noted that even MPO-DNA complexes, while considered the most operationally specific NETosis-related marker among those assessed, are not entirely free from interpretive ambiguity in a salivary matrix. MPO may be released during classical neutrophil degranulation independently of NET formation, and the potential for non-NETotic DNA sources to associate with extracellular MPO in the oral environment cannot be completely excluded. Accordingly, the findings should be interpreted as evidence of NETosis-related neutrophil activation rather than as direct confirmation of NET formation per se.

Cell-free DNA is similarly nonspecific, as it may originate from apoptosis, necrosis, active cellular secretion, oral epithelial cells, bacteria, and other immune cells in addition to NETs. The coordinated elevation across all four markers, including the two more specific ones, supports the overall biological interpretation, but neutrophil elastase and cfDNA should be regarded as contextually supportive rather than independently diagnostic of NETosis. Furthermore, unstimulated whole saliva is a composite fluid receiving contributions from gingival crevicular fluid, major and minor salivary gland secretions, oral epithelial cells, and microbial products; biomarker signals cannot therefore be attributed exclusively to periodontal tissue sources or to a single cellular mechanism.

Moreover, microbiome profiling was not performed, which represents a significant interpretive limitation. Smoking is known to alter the composition and functional profile of the subgingival microbial community, promoting the expansion of anaerobic and dysbiotic species associated with periodontal tissue destruction and suppressing commensal taxa that support microbial homeostasis [42]. Dysbiotic subgingival communities can independently stimulate neutrophil recruitment and activation, promote NET formation, amplify pro-inflammatory cytokine signaling, and enhance oxidative stress responses in the periodontal environment. Consequently, it cannot be determined from the present data whether the observed between-group differences in NETosis-related and oxidative-inflammatory biomarkers primarily reflect direct modulation of host neutrophil and immune responses by tobacco smoke constituents, indirect effects mediated through smoking-induced subgingival microbial dysbiosis, or—most plausibly—a combination of both operating through interacting biological pathways. The absence of microbiome data therefore means that the observed salivary phenotype cannot be attributed exclusively to host-level smoking effects, and that differences in microbial community composition across smoking-status groups represent an uncontrolled biological variable in the present study. Integrated host-microbial analyses that combine subgingival microbiome profiling with salivary NETosis-related and oxidative-inflammatory biomarker assessment in matched populations are a crucial and needed step forward for understanding these contributions.

Another limitation relates to the interpretation of former-smoker status. Time since cessation, previous smoking intensity, and cumulative exposure may vary substantially among former smokers. Although pack-years and cessation duration can be incorporated into statistical models, they may not fully capture individual biological recovery after smoking cessation. Longitudinal cessation studies would be needed to determine whether NETosis markers decline over time after smoking discontinuation and whether such reductions parallel improvements in periodontal stability. A further limitation is that low detectable salivary cotinine concentrations were observed in former smokers. Although these levels were substantially lower than those of current smokers and were interpreted in relation to passive smoke exposure, occasional unreported nicotine exposure cannot be completely excluded.

Future studies should extend these findings in several directions. Prospective cohorts could test whether baseline MPO-DNA complexes, citrullinated histone H3, neutrophil elastase, or composite NETosis scores predict periodontal progression. Interventional studies could evaluate whether smoking cessation reduces salivary NETosis activity and whether this reduction improves clinical response to non-surgical periodontal therapy. Trials of adjunctive host-modulation strategies may also consider NETosis-related biomarkers as exploratory endpoints. In addition, combined salivary, gingival crevicular fluid, microbiome, and metabolomic approaches could clarify whether the smoking-associated phenotype is driven primarily by neutrophil priming, oxidative stress, microbial dysbiosis, impaired resolution of inflammation, or their interactions.

## 5. Conclusions

Current smokers with periodontitis showed a distinct salivary host-response profile associated with higher NETosis-related biomarker levels and a greater oxidative-inflammatory burden compared with former and never-smokers. Salivary MPO-DNA complexes and the composite NETosis-related score were highest in current smokers, suggesting their potential as candidate non-invasive markers of smoking-associated periodontal inflammatory burden, though diagnostic performance analyses and prospective validation are required before any clinical applicability can be established.

Former smokers displayed an intermediate biomarker profile, suggesting partial attenuation of smoking-related inflammatory and NETosis-associated biomarker levels, possibly reflecting biological changes associated with smoking cessation. Smoking exposure, as reflected in salivary cotinine and cumulative pack-years, was positively associated with NETosis and oxidative-inflammatory biomarkers. Higher biomarker levels were associated with greater periodontal severity, supporting the relevance of salivary host-response profiling for periodontal risk assessment in smokers.

## Figures and Tables

**Table 1:** Demographic and periodontal characteristics according to smoking status.

Variable	Current Smoker	Former Smoker	Never-Smoker	*F*	*p*	*η^2^*
Age, years	48.47 ± 5.94 ^a,b^	52.28 ± 8.83 ^a^	47.45 ± 9.67 ^b^	4.98	0.008	0.060
Female sex, n (%)	22 (41.5)	24 (45.3)	26 (49.1)	0.61	0.737	NA
BMI, kg/m^2^	25.73 ± 2.74 ^a^	25.76 ± 3.08 ^a^	25.29 ± 2.37 ^a^	0.48	0.622	0.006
Remaining teeth, n	25.17 ± 2.18 ^a^	25.81 ± 2.05 ^a,b^	26.23 ± 2.37 ^b^	3.09	0.048	0.038
Plaque index, %	42.44 ± 10.92 ^a^	37.45 ± 10.01 ^b^	35.00 ± 11.17 ^b^	6.64	0.002	0.078
Mean PPD, mm	4.47 ± 0.42 ^a^	4.21 ± 0.39 ^b^	3.94 ± 0.34 ^c^	25.72	<0.001	0.248
Mean CAL, mm	5.49 ± 0.49 ^a^	5.07 ± 0.56 ^b^	4.80 ± 0.55 ^c^	22.11	<0.001	0.221
Bleeding on probing, %	52.06 ± 9.28 ^a^	50.92 ± 7.76 ^a^	46.77 ± 8.87 ^b^	5.48	0.005	0.066
Sites with PPD ≥ 5 mm, n	31.26 ± 5.64 ^a^	26.28 ± 6.86 ^b^	20.60 ± 6.86 ^c^	35.94	<0.001	0.315
PISA, mm^2^	1720.53 ± 266.56 ^a^	1551.52 ± 234.98 ^b^	1330.41 ± 223.54 ^c^	34.53	<0.001	0.307

Values are presented as mean ± SD or n (%). Superscript letters denote Tukey HSD post hoc comparisons: groups sharing the same letter do not differ significantly (*p* ≥ 0.05). *F*, F-statistic from one-way ANOVA; *η*^2^, eta-squared effect size. Female sex was compared using chi-square test.

**Table 2:** Distribution of periodontitis stage and grade according to smoking status.

Variable	Current Smokers (n = 53)	Former Smokers (n = 53)	Never-Smokers (n = 53)	Total (n = 159)	*p-*Value
Periodontitis stage					
Stage II	2 (3.8%)	7 (13.2%)	24 (45.3%)	33 (20.8%)	<0.001
Stage III	51 (96.2%)	46 (86.8%)	29 (54.7%)	126 (79.2%)	
Periodontitis grade					
Grade B	6 (11.3%)	40 (75.5%)	42 (79.2%)	88 (55.3%)	<0.001
Grade C	47 (88.7%)	13 (24.5%)	11 (20.8%)	71 (44.7%)	

Note. Values are presented as n (% within smoking-status group). Between-group comparisons were performed using the chi-square test.

**Table 3:** Smoking exposure characteristics and biochemical validation.

Variable	Current Smoker	Former Smoker	Never-Smoker	Test Statistic	*p*
Cigarettes/day during active smoking	15.02 ± 4.97	14.72 ± 4.68	NA	251.85	<0.001
Smoking duration, years	29.36 ± 6.45	17.87 ± 6.99	0.00 ± 0.00	385.00	<0.001
Pack-years	22.54 ± 10.30	13.49 ± 7.23	0.00 ± 0.00	129.16	<0.001
Years since cessation	NA	8.39 ± 4.12	NA		
Salivary cotinine, ng/mL	312.25 ± 76.97	9.72 ± 5.09	3.22 ± 1.39	832.99	<0.001
Exhaled CO, ppm	20.17 ± 4.17	3.75 ± 1.06	2.27 ± 0.74	825.29	<0.001

Data are presented as mean ± SD. Salivary cotinine was assessed in all participants. Years since cessation applies clinically only to former smokers. CO, carbon monoxide.

**Table 4:** Salivary NETosis and oxidative-inflammatory biomarkers according to smoking status.

Biomarker	Current Smoker	Former Smoker	Never-Smoker	*F*	*p*	FDR *q*	*η* ^2^
MPO-DNA complexes, ng/mL	33.52 ± 9.96	26.90 ± 8.38	19.20 ± 7.50	36.22	<0.001	<0.001	0.317
Citrullinated histone H3, ng/mL	14.78 ± 4.21	10.87 ± 3.29	8.12 ± 2.63	50.26	<0.001	<0.001	0.392
Neutrophil elastase, ng/mL	156.29 ± 43.07	133.37 ± 31.25	96.52 ± 23.82	42.54	<0.001	<0.001	0.353
Cell-free DNA, ng/mL	398.93 ± 90.25	312.99 ± 59.14	249.30 ± 51.85	62.56	<0.001	<0.001	0.445
MMP-8, ng/mL	51.67 ± 16.91	44.58 ± 17.29	31.96 ± 9.97	23.16	<0.001	<0.001	0.229
IL-1β, pg/mL	15.42 ± 6.75	13.30 ± 3.68	9.49 ± 3.18	20.71	<0.001	<0.001	0.210
IL-6, pg/mL	5.97 ± 2.74	4.80 ± 1.66	3.28 ± 1.25	24.37	<0.001	<0.001	0.238
TNF-α, pg/mL	7.90 ± 2.35	6.60 ± 2.29	4.99 ± 1.96	23.12	<0.001	<0.001	0.229
8-OHdG, ng/mL	4.26 ± 1.33	3.54 ± 1.30	2.53 ± 0.67	31.00	<0.001	<0.001	0.284
Total antioxidant capacity, mmol/L	0.63 ± 0.07	0.75 ± 0.08	0.82 ± 0.07	101.53	<0.001	<0.001	0.566
NETosis score	0.73 ± 0.37	0.07 ± 0.37	−0.81 ± 0.49	183.46	<0.001	<0.001	0.702
Oxidative-inflammatory score	0.63 ± 0.39	0.08 ± 0.27	−0.71 ± 0.37	198.36	<0.001	<0.001	0.718

Data are presented as mean ± SD of untransformed values. *p*-values and F-statistics are from one-way analysis of variance on raw data. For MPO-DNA complexes and other biomarkers showing significant departure from normality (Shapiro–Wilk *p* < 0.05 in one or more groups), results were confirmed by log-transformed analysis of variance and Kruskal–Wallis testing; all comparisons remained significant and concordant. FDR *q*-values were calculated using the Benjamini–Hochberg procedure. MPO, myeloperoxidase; MMP-8, matrix metalloproteinase-8; IL, interleukin; TNF-α, tumor necrosis factor-α; 8-OHdG, 8-hydroxy-2′-deoxyguanosine; *η*^2^, eta-squared effect size. All secondary biomarker *p*-values were adjusted for multiplicity using the Benjamini–Hochberg false discovery rate procedure (11 tests). All FDR-adjusted *p*-values remained <0.001.

**Table 5:** Post hoc pairwise comparisons for the primary and composite biomarker outcomes.

Outcome	Pairwise Comparison	Mean Difference	95% CI	Adjusted *p*
MPO-DNA complexes	Current smoker vs. Former smoker	6.626	2.640 to 10.612	<0.001
MPO-DNA complexes	Current smoker vs. Never-smoker	14.325	10.338 to 18.311	<0.001
MPO-DNA complexes	Former smoker vs. Never-smoker	7.698	3.712 to 11.684	<0.001
NETosis score	Current smoker vs. Former smoker	0.660	0.469 to 0.851	<0.001
NETosis score	Current smoker vs. Never-smoker	1.538	1.348 to 1.729	<0.001
NETosis score	Former smoker vs. Never-smoker	0.878	0.688 to 1.069	<0.001
Oxidative-inflammatory score	Current smoker vs. Former smoker	0.542	0.383 to 0.702	<0.001
Oxidative-inflammatory score	Current smoker vs. Never-smoker	1.335	1.176 to 1.495	<0.001
Oxidative-inflammatory score	Former smoker vs. Never-smoker	0.793	0.633 to 0.953	<0.001

Mean differences are shown as the first-listed group minus the second-listed group. Positive values indicate higher levels in the first-listed group. Pairwise comparisons were performed using Tukey’s honestly significant difference test.

**Table 6:** Spearman correlations between smoking exposure, biomarker scores, and periodontal severity among current and former smokers.

Exposure	MPO-DNA ρ (*p*)	NETosis Score ρ (*p*)	Oxidative-Inflammatory Score ρ (*p*)	PISA ρ (*p*)	Mean PPD ρ (*p*)
Pack-years	0.366 (<0.001)	0.478 (<0.001)	0.629 (<0.001)	0.516 (<0.001)	0.352 (<0.001)
Cigarettes/day	0.195 (0.046)	0.200 (0.040)	0.255 (0.008)	0.329 (<0.001)	0.166 (0.090)
Salivary cotinine	0.313 (0.001)	0.633 (<0.001)	0.597 (<0.001)	0.312 (0.001)	0.242 (0.013)
Exhaled CO	0.333 (<0.001)	0.613 (<0.001)	0.554 (<0.001)	0.306 (0.001)	0.229 (0.018)

Values are Spearman correlation coefficients with *p*-values in parentheses. PISA, periodontal inflamed surface area; PPD, probing pocket depth; MPO, myeloperoxidase; CO, carbon monoxide.

**Table 7:** Adjusted multivariable regression models for primary biomarker and periodontal inflammatory burden outcomes.

Outcome	Predictor	β	SE	95% CI	*p*
Log MPO-DNA	Former vs. never	0.292	0.065	0.163 to 0.421	<0.001
Log MPO-DNA	Current vs. never	0.573	0.080	0.414 to 0.731	<0.001
NETosis score	Former vs. never	0.776	0.085	0.609 to 0.943	<0.001
NETosis score	Current vs. never	1.507	0.104	1.302 to 1.712	<0.001
PISA	NETosis score	57.939	41.741	−24.543 to 140.420	0.167
PISA	Oxidative- inflammatory score	175.184	48.501	79.344 to 271.023	<0.001
PISA	Plaque index	8.944	1.590	5.802 to 12.086	<0.001

Models for log MPO-DNA and NETosis score were adjusted for age, sex, body mass index, plaque index, number of remaining teeth, periodontitis stage, and periodontitis grade. The PISA model was adjusted for smoking group, age, sex, body mass index, plaque index, and number of remaining teeth. Never-smokers served as the reference category for smoking status. PISA, periodontal inflamed surface area. For log MPO-DNA models, β coefficients represent differences in log-transformed MPO-DNA levels relative to never-smokers.

## Data Availability

The deidentified clinical, periodontal, smoking-exposure, and salivary biomarker data supporting the findings of this study are available from the corresponding authors upon reasonable request. Requests will be assessed in accordance with the institutional ethics approval and applicable data protection regulations. Biological sample records and laboratory output files are retained on institutional password-protected systems. No publicly accessible dataset was generated because the data contain participant-level clinical and biomarker information.

## Supplementary Materials

The following supporting information can be downloaded at: https://www.mdpi.com/article/10.3390/biomedicines14061272/s1: Figure S1: Participant flow diagram. Of 214 individuals assessed for eligibility, 55 were excluded based on pre-specified criteria. The remaining 159 participants were equally allocated into three smoking-status groups (current smokers, former smokers, and never-smokers; n = 53 per group). All enrolled participants completed the clinical examination, saliva collection, and biomarker analysis, and all were included in the final statistical analysis with no missing data. Sensitivity analyses were performed in the Stage III periodontitis subgroup (n = 126) and after exclusion of former smokers with salivary cotinine > 10 ng/mL (n = 135); Table S1: Distributional assessment and sensitivity analyses for salivary biomarker between-group comparisons; Table S2: Salivary NETosis and oxidative-inflammatory biomarkers according to smoking status in the Stage III periodontitis subgroup (n = 126); Table S3: Salivary NETosis and oxidative-inflammatory biomarkers according to smoking status after exclusion of former smokers with salivary cotinine > 10 ng/mL (n = 135); Table S4: Cross-composite Spearman correlations between all ten log-transformed biomarkers.

## Abbreviations

The following abbreviations are used in this manuscript:

8-OHdG	8-hydroxy-2′-deoxyguanosine
ABTS	2,2′-azino-bis(3-ethylbenzothiazoline-6-sulfonic acid)
ANOVA	Analysis of variance
BMI	Body mass index
BOP	Bleeding on probing
CAL	Clinical attachment level
CI	Confidence interval
CO	Carbon monoxide
DNA	Deoxyribonucleic acid
DNase	Deoxyribonuclease
dsDNA	Double-stranded deoxyribonucleic acid
ELISA	Enzyme-linked immunosorbent assay
FDR	False discovery rate
GCF	Gingival crevicular fluid
H3	Histone H3
HS	High sensitivity
HSD	Honestly significant difference
ICC	Intraclass correlation coefficient
IL	Interleukin
IL-1β	Interleukin-1 beta
IL-6	Interleukin-6
MMP-8	Matrix metalloproteinase-8
MMP-9	Matrix metalloproteinase-9
MPO	Myeloperoxidase
MPO-DNA	Myeloperoxidase-DNA complexes
NA	Not applicable
NET	Neutrophil extracellular trap
NETs	Neutrophil extracellular traps
PISA	Periodontal inflamed surface area
PPD	Probing pocket depth
RNA	Ribonucleic acid
RNase	Ribonuclease
SD	Standard deviation
SE	Standard error
TNF-α	Tumor necrosis factor alpha
UNC	University of North Carolina; used here in reference to the UNC-15 periodontal probe
