# Supplementary material for: Salivary NETosis-Related and Oxidative Stress Biomarkers Define a Conventional Cigarette Smoking-Associated Inflammatory Phenotype in Periodontitis: A Cross-Sectional Observational Study
Source: Biomedicines. 2026 Jun 2;14(6):1272. doi: 10.3390/biomedicines14061272 (PMC13297503; doi:10.3390/biomedicines14061272)
Supplement: Supplementary file 1 [file biomedicines-14-01272-s001.zip › biomedicines-4335466-supplementary.pdf]

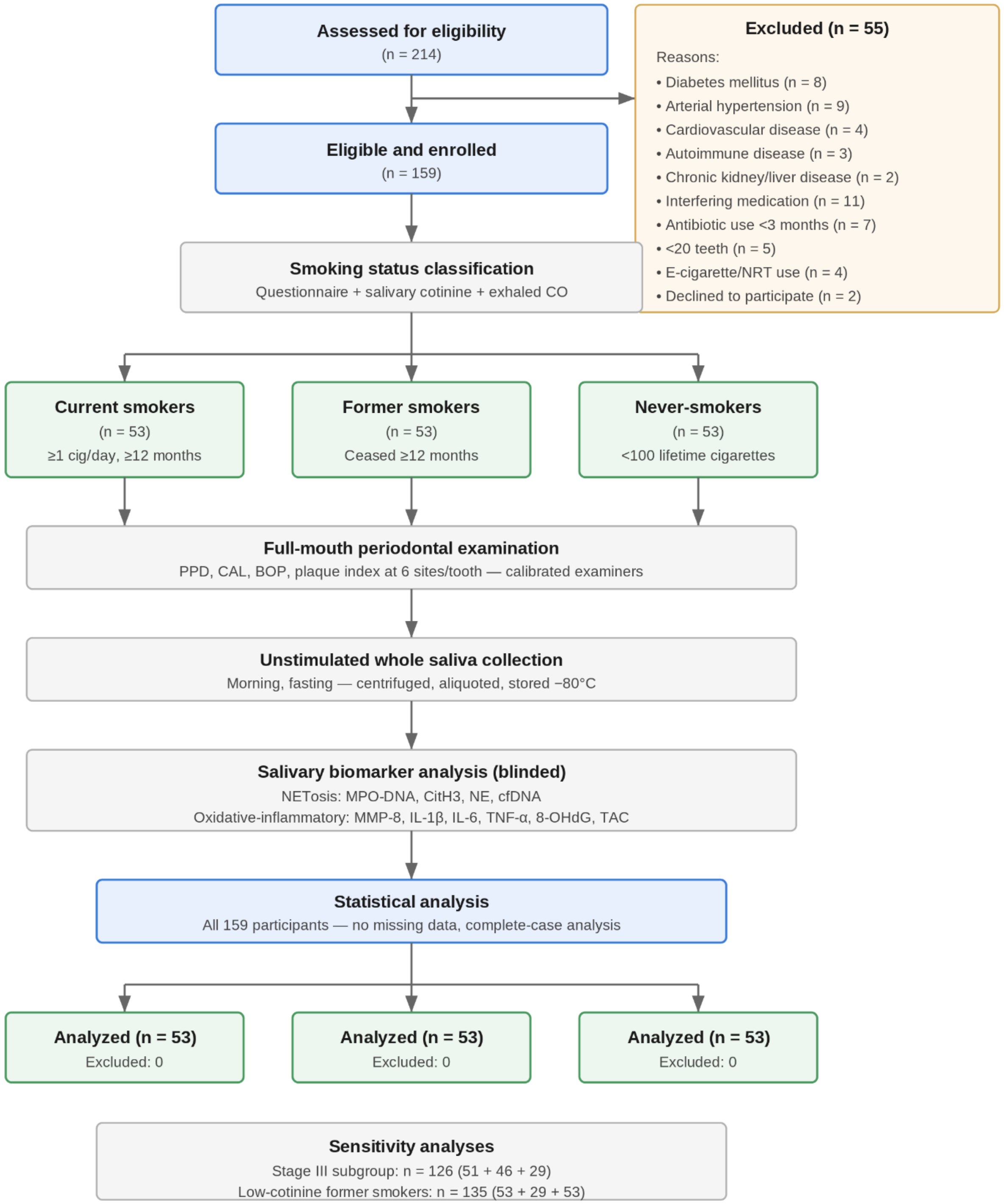

Supplementary Figure S1. Participant flow diagram. Of 214 individuals assessed for eligibility, 55 were excluded based on pre-specified criteria. The remaining 159 participants were equally allocated into three smoking-status groups (current smokers, former smokers, and never-smokers; n = 53 per group). All enrolled participants completed the clinical examination, saliva collection, and biomarker analysis, and all were included in the final statistical analysis with no missing data. Sensitivity analyses were performed in the Stage III periodontitis subgroup (n = 126) and after exclusion of former smokers with salivary cotinine > 10 ng/mL (n = 135).

Supplementary Table S1. Distributional assessment and sensitivity analyses for salivary biomarker between-group comparisons.

	CS	*FS*	*NS*		*F*	*p*	η^2	*F*	*p*	η^2		*H*	*p*	*p*
MPO-DNA complexes, ng/mL	0.025*	0.001*	<0.001*	Yes	36.22	<0.001	0.317	45.89	<0.001	0.370	Yes	58.32	<0.001	0.137
Citrullinated histone H3, ng/mL	0.523	0.013*	0.059	Yes	50.26	<0.001	0.392	52.81	<0.001	0.404	Yes	64.53	<0.001	0.016
Neutrophil elastase, ng/mL	<0.001*	0.010*	0.587	Yes	42.54	<0.001	0.353	52.28	<0.001	0.401	No	66.73	<0.001	0.010
Cell-free DNA, ng/mL	0.097	0.767	0.111	No	62.56	<0.001	0.445	61.07	<0.001	0.439	No	70.81	<0.001	0.000
MMP-8, ng/mL	0.015*	0.005*	<0.001*	Yes	23.16	<0.001	0.229	27.05	<0.001	0.258	Yes	45.24	<0.001	0.006
IL-1β, pg/mL	0.001*	0.454	0.103	Yes	20.71	<0.001	0.210	22.78	<0.001	0.226	Yes	37.61	<0.001	0.000
IL-6, pg/mL	<0.001*	<0.001*	0.075	Yes	24.37	<0.001	0.238	32.65	<0.001	0.295	Yes	43.34	<0.001	0.001
TNF-α, pg/mL	0.311	0.402	<0.001*	Yes	23.12	<0.001	0.229	25.22	<0.001	0.244	Yes	39.00	<0.001	0.379
8-OHdG, ng/mL	0.005*	0.005*	0.016*	Yes	31.00	<0.001	0.284	36.73	<0.001	0.320	Yes	54.52	<0.001	0.004
Total antioxidant capacity, mmol/L	0.414	0.183	0.881	No	101.53	<0.001	0.566	102.32	<0.001	0.567	Yes	90.32	<0.001	0.480

*Normality was assessed using the Shapiro–Wilk test within each smoking-status group. *p < 0.05 indicates significant departure from normality. Raw one-way ANOVA results correspond to those reported in Table 4 of the main manuscript. Log-transformed ANOVA was performed on natural log-transformed values. The Kruskal–Wallis test was used as a non-parametric sensitivity check. Levene's test assessed homogeneity of variance on raw data. All between-group comparisons were significant (p < 0.001) across all three analytical approaches, confirming concordance. CS, current smokers; FS, former smokers; NS, never-smokers; η², eta-squared effect size; H, Kruskal–Wallis test statistic.*

Supplementary Table S2. Salivary NETosis and oxidative-inflammatory biomarkers according to smoking status in the Stage III periodontitis subgroup (n = 126).

Biomarker	CS (n = 51)	FS (n = 46)	NS (n = 29)	F	p	η²
MPO-DNA complexes, ng/mL	33.67 ± 10.12	26.86 ± 8.32	20.94 ± 8.18	19.09	<0.001	0.237
Citrullinated histone H3, ng/mL	14.78 ± 4.28	11.02 ± 3.33	8.58 ± 2.51	29.93	<0.001	0.327
Neutrophil elastase, ng/mL	156.78 ± 43.84	131.62 ± 30.49	97.47 ± 18.90	27.20	<0.001	0.307
Cell-free DNA, ng/mL	404.00 ± 88.18	315.77 ± 58.98	249.16 ± 57.33	45.68	<0.001	0.426
MMP-8, ng/mL	52.27 ± 16.92	44.59 ± 17.17	33.09 ± 10.38	13.70	<0.001	0.182
IL-1β, pg/mL	15.59 ± 6.82	13.15 ± 3.75	10.09 ± 3.10	10.73	<0.001	0.149
IL-6, pg/mL	5.94 ± 2.79	4.88 ± 1.64	3.39 ± 1.36	13.22	<0.001	0.177
TNF-α, pg/mL	7.95 ± 2.37	6.49 ± 2.26	5.09 ± 2.13	15.05	<0.001	0.197
8-OHdG, ng/mL	4.29 ± 1.33	3.57 ± 1.34	2.57 ± 0.70	18.47	<0.001	0.231
Total antioxidant capacity, mmol/L	0.63 ± 0.08	0.74 ± 0.07	0.82 ± 0.08	66.19	<0.001	0.518

Composite NETosis score	0.75 ± 0.37	0.08 ± 0.38	−0.69 ± 0.45	127.65	<0.001	0.675
Composite oxidative-inflammatory score	0.64 ± 0.39	0.09 ± 0.27	−0.63 ± 0.29	137.23	<0.001	0.691

Data are presented as mean ± SD. F-statistics and p-values are from one-way analysis of variance. η², eta-squared effect size. CS, current smokers; FS, former smokers; NS, never-smokers; MPO, myeloperoxidase; MMP-8, matrix metalloproteinase-8; IL, interleukin; TNF-α, tumor necrosis factor-α; 8-OHdG, 8-hydroxy-2′-deoxyguanosine.

Supplementary Table S3. Salivary NETosis and oxidative-inflammatory biomarkers according to smoking status after exclusion of former smokers with salivary cotinine > 10 ng/mL (n = 135).

Biomarker	CS (n = 53)	FS (n = 29)	NS (n = 53)	F	p	η²
MPO-DNA complexes, ng/mL	33.52 ± 9.96	27.71 ± 8.32	19.20 ± 7.50	36.10	<0.001	0.354
Citrullinated histone H3, ng/mL	14.78 ± 4.21	11.21 ± 3.73	8.12 ± 2.63	46.56	<0.001	0.414
Neutrophil elastase, ng/mL	156.29 ± 43.07	136.42 ± 33.78	96.52 ± 23.82	40.53	<0.001	0.380
Cell-free DNA, ng/mL	398.93 ± 90.25	311.65 ± 61.13	249.30 ± 51.85	58.98	<0.001	0.472
MMP-8, ng/mL	51.67 ± 16.91	42.92 ± 18.80	31.96 ± 9.97	22.76	<0.001	0.256
IL-1β, pg/mL	15.42 ± 6.75	13.30 ± 3.86	9.49 ± 3.18	18.88	<0.001	0.222
IL-6, pg/mL	5.97 ± 2.74	5.16 ± 1.63	3.28 ± 1.25	23.89	<0.001	0.266
TNF-α, pg/mL	7.90 ± 2.35	6.88 ± 2.17	4.99 ± 1.96	24.40	<0.001	0.270
8-OHdG, ng/mL	4.26 ± 1.33	3.50 ± 1.17	2.53 ± 0.67	34.54	<0.001	0.344
Total antioxidant capacity, mmol/L	0.63 ± 0.07	0.74 ± 0.06	0.82 ± 0.07	109.37	<0.001	0.624
Composite NETosis score	0.73 ± 0.37	0.12 ± 0.40	−0.81 ± 0.49	172.39	<0.001	0.723
Composite oxidative-inflammatory score	0.63 ± 0.39	0.13 ± 0.30	−0.71 ± 0.37	180.07	<0.001	0.732

Data are presented as mean ± SD. Former smokers with salivary cotinine concentrations exceeding 10 ng/mL were excluded (n = 24 excluded). F-statistics and p-values are from one-way analysis of variance. η², eta-squared effect size. CS, current smokers; FS, former smokers; NS, never-smokers; MPO, myeloperoxidase; MMP-8, matrix metalloproteinase-8; IL, interleukin; TNF-α, tumor necrosis factor-α; 8-OHdG, 8-hydroxy-2′-deoxyguanosine.

Supplementary Table S4. Cross-composite Spearman correlations between all ten log-transformed biomarkers

	log MPO-DNA	log CitH3	log NE	log cfDNA	log MMP-8	log IL-1β	log IL-6	log TNF-α	log 8-OHdG	log TAC (rev)
log MPO-DNA	1.000	0.423	0.462	0.374	0.339	0.241	0.279	0.365	0.318	0.498
log CitH3	0.423	1.000	0.374	0.434	0.322	0.339	0.439	0.353	0.387	0.480
log NE	0.462	0.374	1.000	0.443	0.399	0.346	0.412	0.315	0.399	0.513
log cfDNA	0.374	0.434	0.443	1.000	0.425	0.312	0.324	0.319	0.407	0.517
log MMP-8	0.339	0.322	0.399	0.425	1.000	0.268	0.255	0.198	0.336	0.381
log IL-1β	0.241	0.339	0.346	0.312	0.268	1.000	0.270	0.260	0.320	0.345
log IL-6	0.279	0.439	0.412	0.324	0.255	0.270	1.000	0.126	0.232	0.418
log TNF-α	0.365	0.353	0.315	0.319	0.198	0.260	0.126	1.000	0.361	0.344
log 8-OHdG	0.318	0.387	0.399	0.407	0.336	0.320	0.232	0.361	1.000	0.490
log TAC (rev)	0.498	0.480	0.513	0.517	0.381	0.345	0.418	0.344	0.490	1.000

Values are Spearman correlation coefficients. Items above the diagonal line separate NETosis-related biomarkers (upper-left block) from oxidative-inflammatory biomarkers (lower-right block). CitH3, citrullinated histone H3; NE, neutrophil elastase; cfDNA, cell-free DNA; TAC (rev), total antioxidant capacity reverse-coded.
